# An empirical study of the ‘underscreened’ in organised cervical screening: experts focus on increasing opportunity as a way of reducing differences in screening rates

**DOI:** 10.1186/s12910-016-0143-z

**Published:** 2016-10-06

**Authors:** Jane H. Williams, Stacy M. Carter

**Affiliations:** Centre for Values, Ethics and the Law in Medicine (VELiM), K25, School of Public Health, The University of Sydney, Camperdown, NSW 2006 Australia

**Keywords:** Cervical screening, Underscreened populations, Equity, Justice, Bioethics, Qualitative research

## Abstract

**Background:**

Cervical cancer disproportionately burdens disadvantaged women. Organised cervical screening aims to make cancer prevention available to all women in a population, yet screening uptake and cancer incidence and mortality are strongly correlated with socioeconomic status (SES). Reaching underscreened populations is a stated priority in many screening programs, usually with an emphasis on something like ‘equity’. Equity is a poorly defined and understood concept. We aimed to explain experts’ perspectives on how cervical screening programs might justifiably respond to ‘the underscreened’.

**Methods:**

This paper reports on a grounded theory study of cervical screening experts involved in program organisation. Participants were 23 experts from several countries and a range of backgrounds: gynecology; epidemiology; public health; pathology; general practice; policy making. Data were gathered via semi-structured interview and concepts developed through transcript coding and memo writing.

**Results:**

Most experts expressed an intuitive commitment to reducing systematic differences in screening participation or cancer outcomes. They took three different implicit positions, however, on what made organised programs justifiable with respect to underscreened populations. These were: 1) accepting that population screening is likely to miss certain disenfranchised groups for practical and cultural reasons, and focusing on maximising mainstream reach; 2) identifying and removing barriers to screening; and 3) providing parallel tailored screening services that attended to different cultural needs. Positions tended to fall along country of practice lines.

**Conclusions:**

Experts emphasised the provision of opportunity for underscreened populations to take up screening. A focus on opportunity appeared to rely on tacit premises not supported by evidence: that provision of meaningful opportunity leads to increased uptake, and that increased uptake of an initial screening test by disadvantaged populations would decrease cervical cancer incidence and mortality. There was little attention to anything other than the point of testing, or the difficulties disadvantaged women can have in accessing follow up care. The different approaches to ‘improving equity’ taken by participants are differently justified, and differently justifiable, but none attend directly to the broader conditions of disadvantage.

**Electronic supplementary material:**

The online version of this article (doi:10.1186/s12910-016-0143-z) contains supplementary material, which is available to authorized users.

## Background

Cervical cancer is not an equal opportunity cancer. It is a disease that disproportionately burdens disadvantaged ethnic minorities and women living in poverty in affluent countries, and the poorest women in poor countries [1–4]. Acknowledging population disparities in cervical cancer incidence is not new; cervical cancer has a long reputation of being a disease that disproportionately affects women of low socioeconomic status (SES) [1, 4–7]. Much of this cancer incidence is, in theory, preventable. Screening and vaccination programs aim to prevent or detect the human papillomavirus (HPV) infection that, in rare cases, leads to cervical cancer. The availability and organisation of cervical cancer prevention varies considerably. In some well-resourced countries both primary and secondary prevention methods are widely available and free to women, in others they are easily accessible to those who can pay for them or are insured. In less well-resourced countries access tends to be more limited and also dependent on capacity to pay [8, 9].

Organised cervical screening programs aim to provide optimally efficient and effective screening to a population. Where such programs are in place, women are likely to be informed about, invited, and encouraged to attend cervical screening. Testing in organised programs may be provided at low or no cost, though funding arrangements vary, and efforts are made to provide the service in all geographic regions covered by a program. However while organised programs aim to provide equality of opportunity, equality of outcome is rarely achieved. Disparities in screening uptake also exist; even in countries with equal and free access to screening there have been differences in uptake along SES lines with testing rates decreasing as SES decreases [1, 3, 10]. Despite extensive research on underscreened populations and the barriers to participation those communities face [11–13], discrepancies in uptake remain [8, 14]. Screening those women less likely to be screened – the ‘underscreened’ or the ‘hard to reach’ – remains a stated priority for many cervical screening programs. Disparities do not end with uptake however: rates of follow up of abnormal findings reflect the SES trends that shape the initial screening encounter; that is, the lower the SES the less likely a woman is to receive appropriate follow up care following an abnormal screening result [15–17].

### A focus on uptake and on the underscreened

It is not uncommon for public health interventions to be based on broadly utilitarian reasoning, with the stated goal of optimising aggregated health outcomes at the population level within available resources. This is reflected in the way program successes are reported; in the case of cervical screening a decrease in population cervical cancer incidence over time is a mark of a successful program. Many screening programs also set high targets for screening uptake; in the United States, for example, the goal is that 93 % of women will be screened at least once every 3 years [18]. Where targets are not set at the population level, screening targets at the level of individual primary practice may be set and incentivised [19, 20]. These measures loosely reflect a utilitarian logic: the idea that screening as many women as possible is likely to produce the greatest aggregated population benefit, i.e. the greatest decrease in cervical cancer incidence over time. As is well-recognised, such utilitarian reasoning tends not to focus on who benefits, or how benefit or burden are distributed in the population.

In the case of cervical screening, however, differences in which subpopulations participate in the program are marked. As a result, in the cervical screening literature it is common for increasing uptake and attending to social disparities in uptake to be presented as dual linked goals, for example: “Increased efforts are needed to achieve targets and reduce screening disparities” [18]. However achieving targets and reducing disparities, respectively, may require different approaches and different ways of conceptualising the role of population based screening. The goal of the first is to maximise the number of women screened in a population. The goal of the second is to ensure there are not systematic differences between who is screened and who is not. The usual response in the literature to both of these goals is to promote higher screening uptake, whether in the population as a whole for the purpose of reaching targets or in specific groups who have been identified as underscreened for the purpose of reducing disparity [9, 21–23]. In the cervical screening literature, reducing disparity is often referred to in terms of increasing equity or something like it [9, 24, 25]. In what follows we will argue that the public health ethics literature on equity and related concepts would suggest that increasing equity may require more than simply focusing on uptake.

### ‘Equity’ in health

It is not entirely clear what it means to say that focusing on the ‘hard to reach’ is important in cervical screening, or in public health more generally. Many approaches to public health ethics emphasise something like the interrelated concepts of justice, equity, and/or fairness. Much of the discussion of justice-related concepts and their roles in public health ethics has not necessarily increased clarity; seminal and often cited definitions of, for example, what constitutes inequity in health do not necessarily shed light on meaning. Definitions are often somewhat circular: equity is sometimes described as being about fairness and justice [26] or, for example, “health equity” defined as “social justice in health” [27]. Others claim that social inequality is a key determinant of health and that differential health outcomes that are “socially controllable” are unfair [28, 29]. Problematically, then, such definitions rest on the reader’s own understanding of what these very large concepts –fairness, justice – might mean [26, 30]. Whitehead’s seminal definition says that “[t]he term “inequity” … refers to differences which are *unnecessary* and *avoidable*, but in addition are considered *unfair* and *unjust*” [31] (italics in original). Criticisms of Whitehead’s approach focus on the dynamism of what might be considered necessary and unavoidable. Neither are fixed categories, and both are subject to their changing social and political contexts. Whether or not a condition is considered avoidable can have much to do with how ‘risky’ behaviour is perceived, or the status of technological innovations that might be available to ameliorate difference in some situations but not others [30, 32, 33].

It is these definitions that tend to be used in the public health literature, leaving public health professionals with a strong intuitive commitment to something like equity or justice but no agreed explicit understanding of what that is or what it might entail. Some of the current justice literature attempts to build more robust conceptions of justice in health; much of this revolves around the question of what makes a discrepancy in health outcomes unfair. Preda and Voigt, for example, argue that much of what is called ‘inequity’ in the social determinants of health (SDOH) literature relies on insufficiently examined normative assumptions, and that a focus on health in what they call the “health equity through social change” model detracts from any ultimate goal of increasing social justice [32]. Responses to their paper take mixed stances. Goldberg supports their position, arguing that a lack of attention to the basis for SDOH discourses can lead to empty slogans about health disparities [34]. Others disagree, stating that improved health is a sufficient end in itself and that it is not the role of public health to address social inequities except as they pertain to health outcomes [35, 36]. Preda and Voigt’s response questions the idea of a special role for health that warrants its separate consideration, and they call for more work to be done to strengthen the basis for normative claims of unfairness in the SDOH literature [37].

Fairness, notwithstanding its competing conceptualisations, seems to be considered a salve for health injustice or inequity. Once it has been ascertained that a disparity is unfair, it must be decided what, if any corrective activities need to take place such that unfairness will be avoided or redressed. This involves identifying the end goal of attending to patterns of difference in health. For some the goal lies with the genuine *opportunity* for people to live or act in accordance with their values [38–40]. Proponents of this model hold that different health outcomes can be the result of different values, so opportunities are taken to matter more than outcomes. If everyone has genuine opportunities to access the conditions that produce good health, then equity or something like it is achieved whether or not health outcomes are identical. For others concerned with socially-determined disparities in health, it is *outcomes* that provide a measure of fairness or similar. Outcomes may be in the form of sufficient levels of health [41], the de-clustering of disadvantage [42], a reduction in health distribution gaps [43], or other measures of well-being that provide a measure of fairness or similar.

Maxwell Smith, in a recent examination of justice and health equity, highlights the indeterminacy of how justice is conceptualised [30]. He argues that this lack of clarity carries through into the use of ideas like equity and justice in public health policy making, and calls for empirical studies that examine how public health policy makers understand justice and its related concepts. This is one such study. We interviewed experts involved in establishing, updating or administering organised cervical screening programs in order to answer our research questions: (How) did experts talk about social disparities in cervical screening? What, if any, was considered the best way of overcoming these disparities? Many of the experts we interviewed talked about the need to reduce disparities in screening and the role of equity or something like it in cervical screening. However, as we will show, there was considerable variation in experts’ normative conceptions and evaluations of equity in cervical screening. We describe the different positions experts took on underscreened populations and, in the discussion, contextualise them in the screening pathway and in public health ethics more widely.

## Methods

This study was carried out using grounded theory methods of sampling, data collection and analysis [44, 45]. Organised cervical screening brings together people from many disciplines: pathology; gynaecology; general practice, epidemiology; public health; and public policy. As part of a study into how Australia’s organised cervical screening program came to be the way it is, we purposively sampled experts from these professional backgrounds who were publically identifiable as a result of their work in organising cervical screening in Australia. As data analysis progressed we developed concepts that explained organisation in Australia; we sought to test these against interviews with cervical screening experts working in other countries. We theoretically sampled experts who had been involved in guideline setting primarily in New Zealand because of the relatively similar demographics and health care systems and then further sought out experts from other countries.

Approximately half of the experts we approached agreed to participate in the study. In total we conducted semi-structured interviews with 22 experts and received written commentary via email from one more expert who declined to be interviewed. Permission was given for the contents of that email to be included in the study. Of the 23 participating experts, 14 had worked primarily in Australia, five primarily in New Zealand, and four we characterise as ‘international’ given their breadth of experience. 17 participants had worked in or consulted to cervical screening programs in countries not their own, including programs in North America, Europe, South America, and Asia. Experts had experience of organising cervical screening over a range of time periods (1980s–2010s). Ethics approval was obtained by the Cancer Institute New South Wales Human Research Ethics Committee (HREC/12/CIPHS/46) and the University of Sydney Ethics Committee (#15245).

Interviews were conducted in person and by telephone or Skype by JW and lasted between 35 and 107 min, median 53 min. Telephone interviews have been shown empirically to provide data of comparable quantity and quality to face to face interviews [46]. Interviews were recorded (except for one where the interviewee requested that the interview not be recorded but gave permission for notes to be taken and used as data), de-identified and transcribed verbatim. Data were analysed as we went in order to facilitate comparison with earlier interviews and to help shape question routes. Questions were altered as the study progressed and to ensure each interview was responsive to the experience of the particular participant. A sample question route is available in Additional file 1. JW wrote memos after each interview and again after each transcript was coded, also to facilitate comparison. As we identified something resembling equity as a frequently occurring concept in the data, we began to build a memo around this concept and refined it as we sampled theoretically.

Representative quotes are included in this paper for their illustrative value. Details in quotes that do not affect meaning have been slightly altered to ensure the speaker cannot be identified. Due to the relatively small pool of cervical screening experts in many countries we do not include potentially identifying demographic information such as profession or country of practice alongside the quotes in this paper.

## Results

The central concern for most experts in this study was ensuring that as many women as possible were screened. To this end, they considered that a major benefit of organised screening was its potential to maximise the uptake of cervical screening in the population via the wide distribution of the initial screening encounter. Experts also acknowledged disparities in cervical screening access and cancer incidence. Most experts had an intuitive but undefined sense that it was important to narrow sociodemographic differences in cervical screening and cancer; however this was held more strongly by some experts than others. There were two assumptions behind experts’ talk about screening distribution: 1) that if there was a meaningful opportunity to be screened, women would take it up; and 2) that increased uptake of an initial screening encounter by traditionally underscreened groups would lead to less SES-based difference in screening rates and thus benefit. That is, experts appeared to presume that women would screen if they could, and that higher uptake by underscreened groups would subsequently lead to less cervical cancer in those groups, reducing or removing cervical cancer disparities.

### Experts took one of three implicit positions on what makes a justifiable organised cervical screening program

When experts talked about underscreened populations, they did so with a view to increasing participation in screening programs. They took, broadly, one of three positions on how to best achieve this in order to improve what they usually referred to as ‘equity’. The first position focused on wide geographic availability of ‘mainstream’ screening services, the second on the removal of practical barriers to the access of mainstream screening services, and the third on tailoring screening services to make them more culturally appealing to different sub-populations. We discuss these positions below, along with the geographic patterns we observed in the positions experts took on disparity.

### Position one: Maximise aggregated benefit by making the same services available in all geographic areas

A minority of experts took a loosely utilitarian perspective on the goals of screening, promoting the view that utility could be maximised by ensuring all women had access to screening services. For these experts, screening was about maximising aggregated health via maximising screening in the population within existing parameters. This approach did not presume that absolutely all women would or should be screened. In simple utilitarian reasoning, there is generally an acceptance that failure to benefit a minority, or even harming a minority, is justifiable, as long as aggregated utility is maximised overall. In this case, it was accepted that there would always be a minority of women who would not be screened, and so would not receive any possible benefits of screening. Because this was both seen to be inevitable and thought not to undermine overall utility, it was considered (tacitly) ethically justifiable. It was assumed that there were two types of women who would inevitably not participate in cervical screening. The first were those who were completely estranged from the health care system, as described by Expert 12:

*“Um, I mean, from a clinician’s point of view the cancers we see now are new migrants, um - - - the crazy people, the schizophrenics, the - the ostracised from the health system, the people who’ve got no real connection to the health system, um, I think – I think – I think the cancers that I’m seeing still, no screening program is ever going to impact.”*

The second group was women who were less likely to participate for cultural reasons:

*“If women are not well-informed, or services are not properly available to them, these are things that can and should be fixed. Ultimately, though, we will get to a number of people. I mean, it’s entirely understandable that immigrants – recent immigrants from Pakistan, where the culture is no, you don’t show your private parts to anybody - - - ah, you know, we aren’t going to change them. Ah, it’s – it’s right and proper they’re allowed to keep their, ah, cultural beliefs.”* (Expert 19)

Both of these causes of non-participation were assumed to be relatively intractable. That is, overcoming them would be extremely difficult and thus expensive (carry a high opportunity cost and so undermine overall utility), as well as possibly requiring the violation of other tacit normative commitments such as, for example, respect for cultural difference. Therefore, a justifiable program was one that: *ensured that services were available in all geographic locations, and that standardised information was consistently communicated, without making special efforts to reach women who were highly unlikely to participate.*

### Position two: Reach underscreened populations by removing practical barriers to screening

The majority of experts considered that offering the same service in all geographic areas was not sufficient to engage underscreened populations, and that screening programs had an obligation to provide *effective* opportunities for participation to all women. Of this majority, most experts advocated for identifying barriers to mainstream services and removing them to improve accessibility (Position Two). The basis for this view was that all women do not have the same capacity to access mainstream services and that the opportunity to participate was therefore not the same for all women. Providing effective opportunity to participate in screening meant identifying a range of barriers to access that might be experienced by some women; these tend to be factors such as: cost; fear; or availability of female screeners. Responses to these barriers might involve practical changes, such as cost removal, or informational and educational interventions such as translation of standardised materials into different languages or information sessions for community groups. One expert gave examples of how health system arrangements could inadvertently make screening more difficult for some women than others: *“I think, you know, we need to deal with those accessibility issues that people face … you know, make sure it’s free for everyone and that it can be provided by women … and not prohibiting things like practice nurses being able to do it.”* (Expert 3) Removal of barriers would mean that services were not only available in all geographic areas, but that more women would have a genuine opportunity to access these services, and thus the program would become more ‘equitable’. For this group a justifiable program was one in which: *any woman, regardless of her circumstances, has the same effective access to screening as any other woman, because all identified barriers to accessing mainstream services had been removed for all women.*

### Position three: Reach underscreened populations by culturally tailoring screening services

A third, minority, view was that the program or screening service itself should be tailored by offering separate, different, screening arrangements for identified groups of women in tandem with mainstream services offered to the population. The aim of this approach was to meet the needs of women whose non-participation was for cultural, rather than practical or knowledge-based, reasons. This view was based on the same premise as that used by experts supporting barrier removal, that all women should not only have cervical screening services geographically available, but should also be able to effectively access screening. They agreed that the removal of barriers is necessary to encourage access to *mainstream* services, but contended that this approach was insufficient to ensure that all women could effectively access screening. Instead they argued that particular groups of women had specific needs that could only be addressed by the provision of tailored services that departed from the mainstream. Expert 7 provided examples of how service provision could more meaningfully meet needs of under-screened groups: *“But, ah, they set up a lot of training courses for nurses and for lay smear-takers. And that was a special issue amongst [a particular cultural group] … “Um, the other thing to say is that there was a great anxiety amongst [this group] about a compulsory register. So it was set up so there was a different sort of approach to [their] data.”* Other experts talked about the possibility for underscreened women to access self-administered HPV tests, rather than attend mainstream services. When experts used the word ‘equitable’ in promoting this approach, they meant something like: *a program in which any woman, regardless of her circumstances, has the same effective access to screening as any other woman, because different services are provided in parallel to address the different needs of different groups of women.*

### A fourth view

One expert had a view that differed from all of these positions but was associated with aspects of both Positions One and Three. Expert 14 agreed that screening had to be made culturally relevant before women could be expected to participate, but also agreed with the view that cervical screening was not a service that could necessarily be relevant to the whole population: *“I also think that for some of these people, there will be like this weird experience, where, you know, the children in the community are glue sniffing and there is a high rate of diabetes and chlamydia … equity would say [screening] it should be there but it is probably not at the top of their list of, you know, health concerns”* (Expert 14). This expert advocated for seeing cervical screening as relatively minor part of a bigger public health picture; screening was a ‘nice to have’, but it was primarily the job of public health to attend to health needs that women in vulnerable communities would identify themselves as pressing and currently being experienced. A justifiable program in this outlier view was one where: *other health needs were consistently and reliably met such that preventive care such as cervical screening would come to have relevance to women’s lives.*

### Geographic patterning in expert positions

We noted a pattern in how experts talked about responses to social disparities in cervical cancer and hypothesise that experts’ considerations of equity are formed at least in part by the socio-political discourses that shape public health norms and priorities. The Australian experts we interviewed tended to talk about the difficulties of providing services in remote geographical areas; that is, they were on the whole more focused on the practical difficulties associated with screening access (as is a consideration for all service delivery in Australia) and took Position One or Two. Position Two was the most common and had broad geographical representation. New Zealand experts tended to take Position Three, most commonly in the context of the importance of respecting Māori (indigenous) culture; this position is reflected in that country’s cervical screening policy: *“In practice, a service can be judged to be equitable ‘when people are treated in as fair a manner as possible by ignoring irrelevant differences between them, but taking into account relevant differences.’ In New Zealand there is a diverse range of cultural groups, and cultural factors can be relevant differences. Thus, a screening programme needs to operate from a cultural context that makes sense to participants*” [46, 47]. There was some Canadian support for Position Three, also in the context of talking about indigenous women.

## Discussion

Experts’ talk about underscreened populations and ‘equity’ in the context of cervical screening was explicitly focused on the opportunity to take up initial screening. This is not unexpected – interviewees were cervical screening experts and the topic of interviews was organised cervical screening. A belief in the worth and value of screening as an activity underpinned their work and this was reflected in their views about the importance of raising screening rates for traditionally underscreened or heard to reach groups. In this discussion we situate experts’ talk about reaching underscreened women in the literature and in public health more widely. This paper is an example of empirical bioethics. We consider experts’ views through the lens of the normative literature while also using their accounts to identify gaps in that literature. From this we draw conclusions for how experts’ views and silences can inform thinking about ‘hard to reach’ groups in cervical screening.

### Situating experts’ views in the literature

Smith calls for empirical work that explores public health practitioners’ normative assumptions about what might constitute health equity [30]. Our analysis shows that cervical screening experts took three alternative positions on what a just cervical screening program looked like. Two of these positions emphasised reducing differences in participation rates among different population groups by targeting those groups for special treatment. Special treatment was intended to lift screening rates in typically underscreened groups to bring them more in line with general population rates. Here we look at the positions in turn.

In an ‘maximise aggregated benefit by making standardised services available in all geographic areas’ scenario, a screening program had an obligation to meet certain conditions universally—especially a standardised geographically accessible service and the provision of adequate information—but was not obliged to screen all women. These experts provided justifications for their position. First, some women had more immediate health concerns, or cultures or values that contraindicated vaginal examinations, and so cervical screening might quite reasonably be seen as irrelevant by them. Taking measures to encourage these women to be screened would involve the commitment of significant resources; persuading truly recalcitrant non-screeners to participate in screening is an expensive proposition, and so has opportunity cost implications. Secondly, it was not simply a question of unnecessary expense but also one of cultural appropriateness – if a woman had a deeply held reason for not participating that was based on cultural or religious belief then it was not the role of the program to interfere with those beliefs.

Because the experts holding ‘maximise utility’ views considered a certain amount of non-participation in screening programs to be inevitable, they viewed special efforts to reach underscreened populations as largely futile. From this perspective, arguments against special treatment on the grounds of cost implications and cultural appropriateness have some credibility. Cervical screening that uses high-quality cytology is expensive, particularly in organised programs such as Australia’s that encourage screening more frequently than every 3 years or from a young age [48]. Additional costs to the program may deem it cost-ineffective. To the issue of cultural intractability, many liberal traditions, including some associated with utilitarian reasoning, would recognise the moral importance of not coercing citizens to act against their own values, even if that reduced the utility afforded by a public program such as cancer screening. This would ordinarily be argued on some version of the utility and/or fundamental moral importance of the liberty of individual citizens [49] (Ch6). With that said, justifications for providing a service and information that are known to appeal to a majority but not an identifiable minority do not respond adequately to the following problems.

Cervical cancer disproportionately burdens already-disadvantaged women. This means that certain people in society, by virtue of their cultural or socioeconomic position, are less able to avoid a higher risk of cervical cancer. Arguing the justifiability of a screening program from a moral position that is largely disinterested in distribution seems to inadequately respond to the very character of cervical cancer, and thus seems to be problematic. If we accept that cervical cancer is a burdensome and usually avoidable disease that disproportionately affects the least well off, *and* that we have good reasons to prefer interventions that are likely to benefit the least well off more than the most well off [41] (ch6), then a justifiable cervical screening program is likely to specifically attend to the needs of the populations that are most likely to benefit. What is not clear is what the resulting program might look like. We now turn to the strengths and shortcomings of the approaches favoured by Positions Two and Three.

The second approach, identifying and removing barriers that are known to undermine access to screening, is an attempt to address the systematic nature of the disparities seen in screening uptake. The justifiability of barrier removal as means of improving equity, or something like it, depends to an extent on what the goal of the intervention is considered to be. Alternative goals could be, for example, 1) to provide meaningful opportunity to participate in screening regardless of background; or 2) to narrow SES-based disparities in screening participation or cancer outcomes. There is a tension between opportunity and outcome here. Experts’ talk focused on the importance of women being able to access screening irrespective of their circumstances, implying an opportunity focus.

As previously shown, Position One assumes it is acceptable to exclude some women from opportunities to participate in a program ostensibly designed to benefit the population of women. The strategy of removing barriers implicitly rejects this view, seeking to remove those things that impede opportunity (such as payment, being examined by a man, or not speaking English). This seems intuitively attractive. Ensuring opportunity in this way, however, does not appear to reliably change the uneven distribution of cancer.

If barrier removal was effective in improving participation, and if this participation had an effect on cancer incidence, it should be possible to demonstrate reduction of cervical cancer incidence and mortality in targeted populations over the longer term when barriers are removed. That evidence, however, is not available: the literature on barrier removal instead reports on whether removing barriers increases screening uptake in targeted populations. Unfortunately, the majority of studies do not show evidence of effectiveness by an increased uptake measure [50–53]. Note here that we are not claiming that barrier removal does not decrease cancer incidence or mortality. Rather, we are claiming that, as yet, the literature has not clearly demonstrated that barrier removal decreases incidence or mortality. The evidence that exists comes from myriad small, population-specific evaluations which are unlikely to be included in aggregated studies, making conclusions difficult to draw. Studies that look at the screening pathway are needed. It is not yet clear the extent to which increased access leads to increased uptake, whether that increased uptake correlates with follow up, or the extent to which diagnoses stemming from that follow up are adequately treated.

If this view of the evidence is accepted, it presents a challenge for the view that opportunity to screen should be the most important indicator of a just program. If equitable opportunity to screen is to be accepted as the indicator of whether a program is just, then one of two things would be needed. First, the opportunity position could be supported by evidence that the removal of barriers is in fact providing women with a real opportunity to consider whether screening is right for them. It is plausible that women may be presented with a meaningful opportunity, decide that screening is not right for them, and decline the offer. This could conceivably be a possible explanation for unchanged uptake following barrier removal interventions. However, evidence of this is currently lacking. Second, in the absence of such evidence, the opportunity argument would need to be supported by better reasoning about why opportunity should matter, if that opportunity appears not to influence uptake, and the ultimate goal of the program is uptake.

This brings us to the third approach, changing screening services to better appeal to specific underscreened groups. This approach recognised that in some instances the service offered to the population was not a service that some groups of women would use for cultural reasons, irrespective of attempts to make it easy to use. This third group of interviewees considered providing services that are relevant to all sub-populations to be a political and moral imperative. It should be noted that the majority of experts taking this view emphasised the cultural needs of indigenous women in particular. In this approach the *means* of achieving higher uptake or narrowing the gap in screening rates (cultural tailoring) was not just an instrumental good. It also had intrinsic value, contributing to self-determination. Position Three experts thought this valuing of culture made a program more justifiable.

Catriona Mackenzie has defined self-determination as follows. *“Self-determination involves having the freedom and opportunities to make and enact choices of practical import to one’s life. … Opportunity conditions [for self-determination] specify the kinds of opportunities that need to be available to agents in their social environments for them to have choices about what to value, what to be, and what to do*” [54] (p17). Public health interventions that take cultural specificities into account seem more likely to promote the circumstances necessary for the realisation of self-determination because they indicate that difference is respected and valued, and they seem likely to be developed in conjunction with or by representatives of the groups in question [55]. Self-determination is intrinsic to conceptions of health and well-being in indigenous populations [56–58] as well as being an identified component of autonomy more generally [54]. It is not clear whether or how well cultural tailoring increases uptake of screening services, because of the way data are recorded or made available for analysis. As such, the same arguments made around tensions between assessing opportunity, outcome and uptake for Position Two, above, hold here too. Where an analysis of the justifiability of Position Three could differ is in considering the contribution its methods or basis in cultural respect might make. Arguably, consultation with marginalised minorities and any subsequent addition of (non-mainstream) culturally specific practices to a population service seems likely to produce goods other than increases in opportunity, screening uptake or disease prevention. Position Three was based on at least some degree of appreciation for cultural variation and political will to value that variation. An additional, and difficult to measure, outcome may be the good which accrues to a marginalised population as a result of this recognition.

It is not only indigenous peoples who experience cultural barriers to screening. Others, particularly immigrant groups, are also systematically disadvantaged. Tailoring screening for these groups, however, was not discussed in any depth in our interviews. In the literature, HPV self-testing is often suggested as a method of overcoming cultural barriers that are connected to modesty or discomfort more generally with vaginal examinations [59–62]. This approach, prima facie, should have potential in providing a culturally acceptable alternative. However, if it is introduced without consultation with traditionally underscreened groups, as sometimes appears to be the case [63], self-determination benefits seem likely to be less.

### Situating cervical screening in public health

Experts’ talk about reaching the underscreened focused on the provision of effective opportunities to participate in screening programs for all women. There are a number of assumptions that underpinned their positions: 1. that preventive health measures could be made relevant and appealing to all women; 2. that effective opportunity to be screened would convert to uptake; and 3. that increase in the uptake of an initial test would convert to less disparity in cervical cancer incidence and mortality. We discuss each of these in turn.

#### Can cervical screening be made relevant to everyone?

There was a broad inclination, as Positions Two and Three describe, towards attempts to make cervical screening effectively accessible or relevant by removing barriers to participation or providing alternative screening services. It is likely, however, that for some women it might not be identifiable practical or cultural misalignment that prevents them from being screened but chaotic lives or immediate health needs that make preventive care seem immaterial, irrespective of attempts to make it so. Sufficientarian models of justice suggest that we should ensure that all members of a society meet a threshold level of wellbeing [41, 64], justified largely by the idea that it is not the gap between poverty and affluence that is troubling, but the conditions of poverty themselves. Such arguments suggest that a baseline level of wellbeing may be necessary before it is possible for cervical screening, or health screening generally, to make sense as an offering. This approach reflects the position taken by the outlier expert, the fourth view described in the results section. Arguably, a public health service, broadly conceived, might have some obligation to support women to reach that threshold level of wellbeing before it tries to offer them screening. If this is accepted, it may not be screening uptake or less screening disparity *per se* that should be measures of health equity. Rather, the achievement of a general level of wellbeing in the lives of all women, such that the opportunity to participate in screening seems relevant to them, may be a better indicator of health equity [65].

#### Opportunity and uptake: the obligation to screen

The literature reports that members of the public may believe there is an obligation to access available cancer screening services [66, 67]. Because the success of screening programs is measured in part by uptake, such that financial incentives are offered to screeners to reach particular screening targets, screening is likely to be communicated to the public in a persuasive manner [68]. When programs have an implicit goal of maximising utility, all participants are imagined to be roughly identical and offering standardised services is seen to be the most efficient and so the most utility maximising. Women in traditionally underscreened groups seem more likely to perceive that service as irrelevant to them, perhaps because they are the most likely to be different than the imagined “standard” woman that these programs are targeting. We can speculate that as particular groups of women are targeted via practical or cultural avenues, a shared perception of obligation to screen may increase. This could be for a number of reasons. 1) Screening programmers could have a stronger desire to make sure that money spent on tailored screening is not ‘wasted’ and might promote services accordingly in the belief that participation is important for both women and the public purse. 2) Services may seem more relevant and more appealing to the groups they target, creating a new shared perception of screening as a social norm. 3) Targeting may also stimulate a sense of reciprocity – if usually disenfranchised groups are offered a special service they may feel a sense of owing something in exchange for the efforts made to include them, or in the hope other types of offers might be made.

There are two conflicting ethical imperatives at play with expending special efforts to particular populations to provide meaningful opportunity to participate in screening. A heightened sense of obligation to undergo screening may be problematic when it comes to meeting conditions for consent because it may undermine the ideal of voluntary choice on the part of consumers that is intended to underpin informed consent [69, 70]. However as we have discussed, a program that is not perceived as accessible or relevant to certain sections of the population is also morally difficult to justify, particularly when it is least relevant to the very people it is most likely to benefit. This tension is unlikely to be resolvable but it might be mitigated by closely attending to how opportunities are communicated. One way of supporting valid consent for screening is known as the ‘consider an offer’ approach [70]. It suggests being explicit about the benefits and harms of a test and the appropriateness of that test for the individual in question. It also recommends being explicit about the organisation behind the testing – who is making the offer? Do they have anything to gain from people’s participation? – and communicating non-compulsion and the availability of further information if it is desired. While the ‘consider an offer’ approach seems likely to support consent for screening there is also the chance that, given the explicit option, women will decide not to participate, which remains a difficult tension for screening programs.

#### Uptake and cancer: the Pap test in the screening pathway

As cervical screening needs contextualising in public health, so does the Pap test (or HPV DNA test, or other initial screening test) need to be seen in the context of the wider screening pathway. While special attention to traditionally underscreened groups might increase uptake of an initial screening encounter, that test can only indicate normal or heightened risk. For that risk to be reduced there must be investigation and, where indicated, treatment. Women who are unlikely to participate in mainstream screening are also less likely to take up recommended diagnostic services and treatment, indicating perceived or actual barriers to participation in some screening programs that go beyond the initial test [15–17, 60]. Therefore increasing screening uptake and the identification of increased risk do not necessarily mean removal of that risk, or improved cervical cancer outcomes. Yet the focus of many organised screening programs, and all of the experts we interviewed, was participation in the initial screening encounter only, which means significant effort and resources put into a test that may produce distress rather than health. It is not clear why experts did not talk about the full screening pathway in interviews, but the same tendency is true of this literature. Barriers to initial screening tests receive full and frequent attention; socially-based discrepancies in follow up of women with abnormal results much less so [16]. Neglecting to talk about the full screening pathway may be just as responsible for the ongoing SES-based disparities in the distribution of cervical cancer as discrepancies in screening uptake.

### What this paper adds to the justice literature

Our analysis shows that experts working on developing and implementing cervical screening programs tend to agree, prima facie, that there is an ‘equity problem’ with cervical screening and cervical cancer, but also tend to disagree on whether or how to address it. We began this paper with the claim that much of the public health literature regarding equity and justice is somewhat circular, and may be unhelpful for people involved in thinking practically about situations involving ‘social’ health differences. Cervical screening is a clear example of ‘social’ health difference: the same patterns of disadvantage in cervical cancer incidence appear to be replicated to a greater or lesser extent across many populations. We have shown that conceptions of what made a program justifiable varied across experts, with quite different consequences for the practical shape of the resulting program. This variation suggests a lack of connection between the literature on equity and justice and the everyday work of screening experts. We would speculate that this may be because the literature does not currently offer easily understood or applicable definitions of these concepts. It is also possible that other, perhaps less conceptually robust, resources offer a quicker and easier apparent solution to the ‘equity problem’. In either of these cases, it is not clear that fine tuning an ideal theory of justice would assist experts involved in program development. Their approaches to what made a justifiable program were deeply contextualised in the programs they had worked on and the patterns they had seen or deduced. It might be that, for the purposes of both public health ethics and public health practice, working out how notions of equity and justice work in a practical sense for a particular case is a more important task than ideal theorising [71].

## Conclusions

There was strong support among the experts we interviewed for targeting underscreened groups in order to provide better opportunities for screening; the same support is found in the cervical screening literature. Broadly, it is assumed that providing meaningful *opportunity* to participate in screening will translate to *actual* participation in screening and, presumably, improved outcomes in cervical screening incidence and mortality for traditionally underscreened groups. There is, however, no clear evidence of such outcomes. As a result we are left with an intuitive sense that increased opportunity is good but no clarity about whether that alleged good makes any difference to women’s lives. Different approaches to ‘improving equity’, as it was described by participating experts, are differently justified, and differently justifiable, but none focus adequately on the big picture. If cervical cancer is strongly associated with disadvantage, but screening is inevitably irrelevant to the most disadvantaged, then the public health program of which screening is part should arguably work to eliminate those conditions that mean screening cannot even be considered. Programs might also attend to the way screening is communicated to make sure that increased attention to some groups does not translate into an increased sense of obligation to be screened. Finally, unless there is attention to the full screening pathway it is difficult to see how providing even a genuine opportunity to take up an offer of an initial screening encounter will lead to improved cervical cancer outcomes.

## Additional file

Additional file 1: Sample interview question route. (PDF 63 kb)

## Abbreviations

HPV: Human papillomavirus
SES: Socioeconomic status
