# Supplementary material for: An empirical study of the ‘underscreened’ in organised cervical screening: experts focus on increasing opportunity as a way of reducing differences in screening rates
Source: BMC Med Ethics. 2016 Oct 6;17:56. doi: 10.1186/s12910-016-0143-z (PMC5053126; doi:10.1186/s12910-016-0143-z)
Supplement: Additional file 1: — Sample interview question route. (PDF 63 kb) [file 12910_2016_143_MOESM1_ESM.pdf]

Sample interview questions

Questions were tailored towards experts' experiences and involvement (which were known ahead of time due to their public positions).

- Can you tell me about your involvement in cervical screening?
- Can you tell me the story of the shift in cervical screening from an opportunistic test to an organised program?
- What do you think prompted the shift? What were the specific objectives? What do you think about those objectives?
- What were the issues on which the 2005 NHMRC process hinged? What triggered the assessment of CIN1 treatment?
- What lessons might the current renewal process take from the processes of setting the program up and developing the guidelines for treatment?
- Were there any difficulties in implementation?
- Do you think the program has achieved what it was set up to do?
- What were the benefits of organisation?
- Were there any downsides to organising screening?
- Did the program change after it was put into place, and if so why?
- How did the shift to the modified Bethesda system happen? Why was that system chosen?
- Why were the registers set up the way they were?
- Was communication with women part of the planning process? At the Commonwealth or state level?
- What do you think has been the legacy of the "unfortunate experiment" in New Zealand, if any, for (Australian) cervical screening? (removed after initial interviews)
- If you could design the program however you wanted and funding wasn't an issue, what would it look like?
- What do you think will happen with the screening program?
- Some people think the cervical screening program might inevitably be discontinued if the HPV vaccine does what we hope it will do. What do you think about that?
- What conditions do you think would need to be met before the population screening program was discontinued?
